# Direct Evidence of Adult Aedes albopictus Dispersal by Car

**DOI:** 10.1038/s41598-017-12652-5

**Published:** 2017-10-24

**Authors:** Roger Eritja, John R. B. Palmer, David Roiz, Isis Sanpera-Calbet, Frederic Bartumeus

**Affiliations:** 1 CREAF, Cerdanyola del Vallès, Spain; 2 Servei de Control de Mosquits del Consell Comarcal del Baix Llobregat, Sant Feliu de Llobregat, Spain; 3 Centre d’Estudis Avançats de Blanes (CEAB-CSIC), Girona, Spain; 4 Universitat Pompeu Fabra, Barcelona, Spain; 5 MIVEGEC (Infectious Diseases and Vectors: Ecology, Genetics, Evolution and Control), Institut de Recherche pour le Développement (IRD), Montpellier, France; 6 ICREA, Institut Català de Recerca i Estudis Avançats, Barcelona, Spain

## Abstract

Whereas the Asian tiger mosquito (*Aedes albopictus*) has low active dispersal capabilities, its worldwide colonization has been rapid. Indirect evidence and informal reports have long implicated passive transportation in cars, but this has not previously been studied systematically given the difficulties of real-time roadside surveys. Here we report the first sampling study confirming that adult tiger mosquitoes travel with humans in cars and enabling us to estimate the frequency of these events. We combine the results with citizen science data to model the car-facilitated dispersal of *Aedes albopictus* at a nationwide level. During the summer of 2015, we sampled 770 cars in north-eastern Spain, discovering 4 adult female tiger mosquitoes that had entered cars prior to sampling. Our Bayesian model suggests that of the 6.5 million daily car trips in the Barcelona metropolitan area, between 13,000 and 71,500 facilitate tiger mosquito movement, and that Barcelona is the largest source of inter-province tiger mosquito transfers in Spain. Our results are supported by expert-validated citizen science data, and will contribute to better understanding the tiger mosquito’s invasion process and ultimately lead to more effective vector control strategies.

## Introduction

Global trade and travel have accelerated the spread of invasive disease vector mosquitoes, with serious implications for human health^1–4^. Successful invasive mosquitoes are able to exploit human transportation channels and thrive in human-dominated habitats through diapause, desiccation-resistant eggs, and the ability to develop in small, man-made containers^5^. Surveillance of transported mosquitoes was focused for many years on international airports due to evidence, including inflight captures, of *Anopheles* females travelling and spreading malaria by airplane^6^. Recently, attention has shifted to the rapid spread of *Aedes* vectors^3,7^, including the Asian tiger mosquito, *Aedes albopictus*, which has now established itself in a large area on all continents except Antarctica, in both tropical and temperate environments^8^. Current tiger mosquito monitoring and surveillance efforts carried out in Europe include innovative citizen science approaches^9^. Citizen science facilitates new discoveries of *Aedes* mosquitoes well beyond the expected invasion range^10,11^, and combined with locally deployed traditional entomological surveys, citizen science makes country-wide monitoring possible^12^.

The tiger mosquito is a threat to human health because it is a vector of dengue, chikungunya and Zika and a potential vector of more than 20 other pathogens^13–16^. Its spread also has important environmental and economic costs, the magnitude of which have yet to be fully appreciated^17^. The tiger mosquito’s invasive efficiency is paradoxical because females of this species are capable of active dispersal over only short ranges^18–20^. As with other *Aedes* vectors, the spread of the tiger mosquito has been linked to global shipping routes and road networks, but much remains unknown about the human role in the dispersal mechanism.

Much like other biological invaders, the tiger mosquito appears to follow a stratified dispersal pattern, suggesting that long-distance transport mechanisms are very different than short-distance ones^21,22^. Long-distance jumps are mainly driven by accidental mass transportation of immature mosquitoes, in particular desiccation-resistant eggs, embedded with merchandise (primarily used tyres, but also lucky bamboo, *Dracaena spp*, and other goods). This focuses species introductions on particular distant areas to which large populations are transferred–likely on a repetitive basis–enhancing the probability of establishment success. This is a key dispersal mechanism and the principal hypothesis for the tiger mosquito’s inter-continental dispersal, as from Asia to the United States, or from the United States to Europe^22,23^. Within countries, indirect evidence suggests dispersal of immature tiger mosquitoes is linked to the commerce of scrap tyres via interstate highway networks^24,25^. During recent years, there has also been increasing discussion of the passive dispersal of adult tiger mosquitoes in private vehicles at both local and medium-range scales^26–29^, which could partly account for observed spreading patterns.

The first detection of *Ae*. *albopictus* in Spain was in Sant Cugat del Vallès, a municipality that contains a major highway junction but no wholesale tyre shippers or other obvious source of mass infestation^30^. The team documenting this detection noted that the introduction of adults in cars or lorries was possible but had not been investigated^30^. Twelve years later, the latest reports on the geographic spread of the species^31^ highlight that major roads run through the present distribution area of *Ae*. *albopictus* ^12^.

To our knowledge, no quantitative evaluation of adult *Ae*. *albopictus* car transport has previously been performed. Instead, this topic has previously been addressed in only general and uncertain terms. For instance, Scholte & Schaffner^26^ note “strong indications that transportation of *Ae*. *albopictus* by car and/or trucks occasionally happens” and that local or short-distance dispersal “is probably based on passive transport of adult mosquitoes in cars and trucks”. Medlock *et al*.^29^ state that *Ae*. *albopictus* movement via public or private ground transport “has been suggested as the main route of dispersal along highway systems”, and Šebesta *et al*.^32^ state that “the most important mode of long-distance dispersal of *Ae*. *albopictus* in Europe in the last decade seems to be transportation by ground vehicles (i.e. lorries, cars, caravans) from southern Europe”. Finally, Flacio *et al*.^27^ and Werner and Kampen (2015)^33^ based their field work design on the assumption of the passive introduction of adults by road in Switzerland and Germany, respectively.

In fact, this knowledge gap is not limited to tiger mosquitoes: There appear to be no scientific works documenting the transportation of any mosquito species by car. As with the tiger mosquito, there is only speculation about the car transportation of other species. Lounibos^3^, for example, notes that the 1939–40 dispersal of *Anopheles gambiae* in Brazil “may have been assisted by car, train or boat”.

Unlike long-distance dispersal, car hitchhiking is an unpredictable dispersal mechanism with no specific directionality, presumably involving very few individuals at a time. While these individuals are assumed to have very low survival chances as adults, the mechanism may nonetheless have important effects given the massive car fluxes involved. This hypothesis is supported by mosquito detections in ovitrap surveillance programs in vehicle rest areas (parking areas, gas stations) along major transportation routes, including highways in Switzerland, France, Panama and elsewhere^21,27,34,35^. Furthermore, for both *Ae*. *albopictus* and *Ae*. *japonicus*, this hypothesis is supported by dispersal models based on comparative genetic diversities^36,37^.

**Figure 1:**
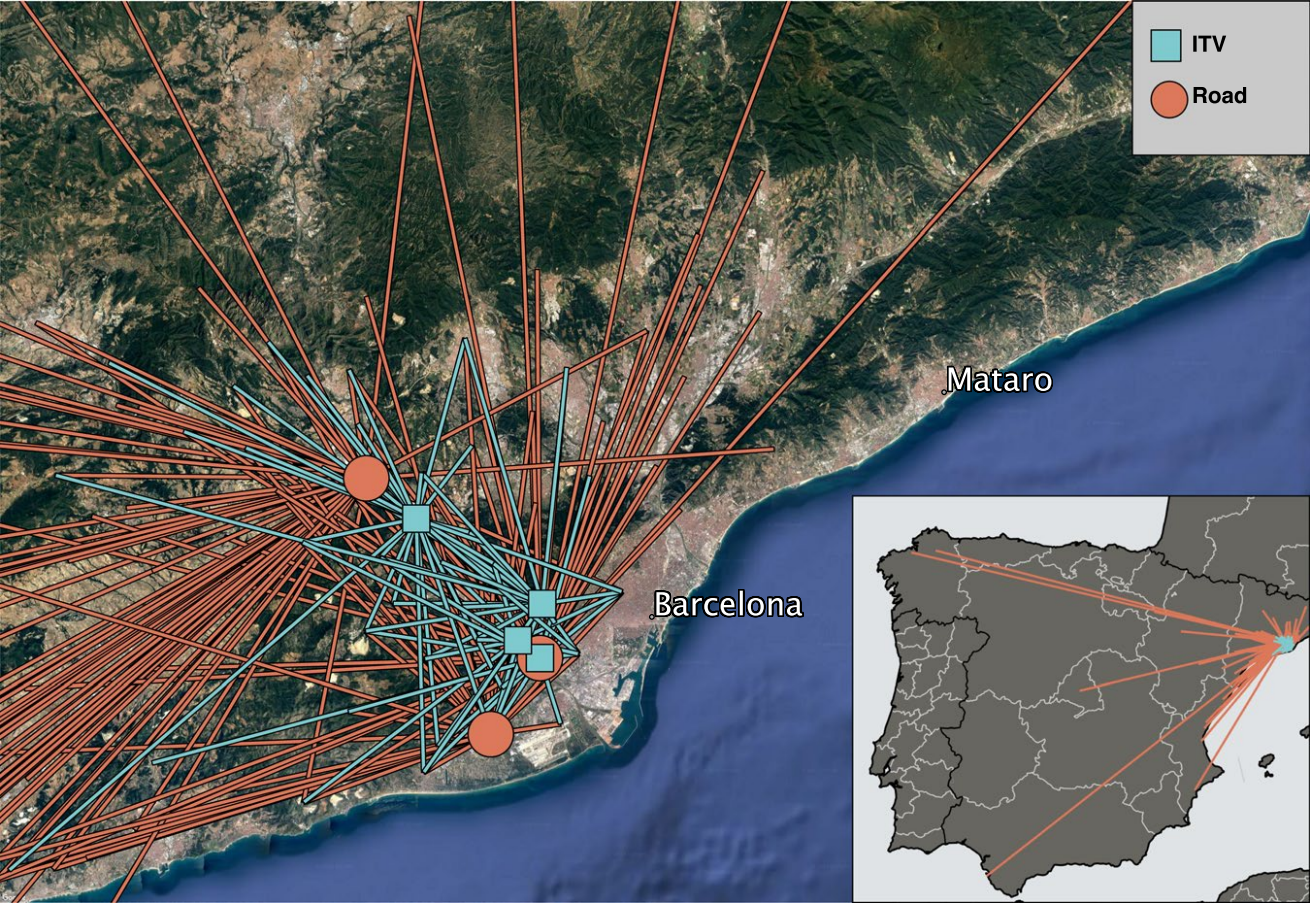
Sampling locations and approximate trip origins in Barcelona area (main map) and Spain (inset). Lines schematically indicate trips to sampling locations at ITVs (blue) and road stops (orange). Lines originate in centre of reported municipality for each trip. Satellite map image from Google: Imagery ©2017 TerraMetrics. Inset vector map made with Natural Earth version 2.0.0. Maps made using Quantum GIS version 2.18 (http://www.qgis.org/).

To confirm and to quantitatively assess this phenomenon, we implemented a random sampling protocol, using an entomological vacuum to check for mosquitoes in cars in the Baix Llobregat area, near the city of Barcelona in Catalonia, Spain. After a preliminary test of sampling efficiency (see Methods), we sampled cars on the road in collaboration with the Catalan Police Department, and at Technical Vehicle Inspection stations (ITVs) in collaboration with the Catalan Department of Industry and the inspection services company Applus+. (ITVs are the places where car compliance with safety and pollution regulations is officially certified; they are similar to inspection stations in most other EU countries). Mosquito detections were recorded together with responses to a driver survey about each car’s starting location and trip conditions. The road sampling provided broad information on vehicles engaged in medium-distance trips with a variety of destinations, while the ITV sampling mostly involved cars at their final destination after very short-distance trips (Fig. 1). We combine the detection and survey results with data on commuting patterns from Spain’s Active Population Survey, and with expert-validated citizen-science data on human-mosquito encounters, in order to corroborate results and estimate potential inter-province flows of tiger mosquitoes in Spain.

## Results

### Evidence from Car Sampling

The results for the car sampling are summarized in Supplementary Table 1. Overall, almost half of the sampled cars had spent the previous night on the street. The mean trip time was much shorter in the ITVs than on the road, as expected because these facilities are widely distributed near residential areas for convenience. Drivers also declined to have their cars vacuumed much more frequently in the ITVs than on the road (Supplementary Table 1).

**Figure 2:**
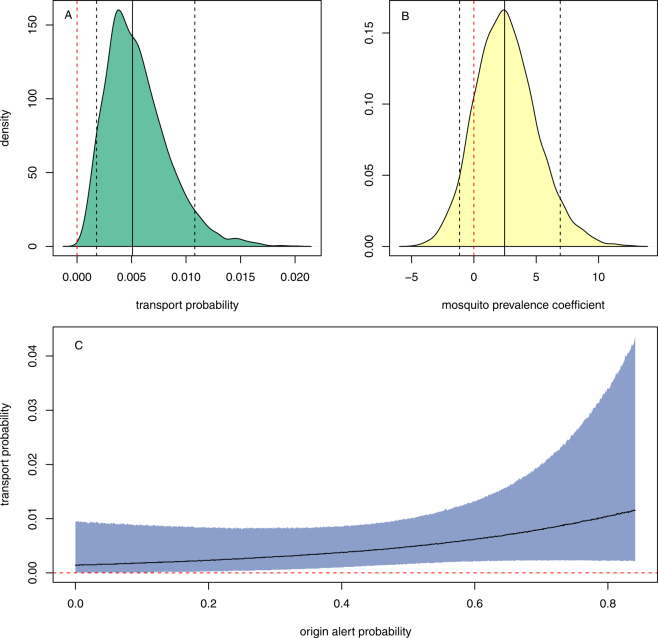
(**A**) Posterior predictive density of the tiger mosquito car-transport probability based on the naive model (no covariates). Solid black vertical line shows median, dotted black vertical lines show bounds of 90% credible interval, and dotted red vertical line marks zero. (**B**) Posterior density of origin alert probability coefficient in car transport model. Solid black vertical line shows median, dotted black vertical lines show bounds of 90% credible interval, and dotted red vertical line marks zero. (**C**) Posterior predictive interval across range of modelled tiger mosquito alert probabilities. Grey area shows 90% credible interval, black curve shows median, and dotted red line marks zero.

A total of 6 adult female mosquitoes were captured but only 4 were considered valid tiger mosquitoes for purposes of this study. Of the other two, one was identified as a *Culex pipiens* specimen, not a tiger mosquito, and the other appeared to have entered the car at the time of sampling and, thus, not to have actually travelled in the car. (Additional insects were also captured but not recorded or retained given the need to avoid unduly delaying each car). Each of the 4 valid tiger mosquitoes was found in a different car and 2 were dead (Supplementary Tables 1–2). Thus, of the 770 cars sampled, 0.52% were determined to be transporting tiger mosquitoes (dead or alive). Modelled using a logistic regression with no covariates and a weakly-informative prior, the estimated posterior distribution for the probability of a car transporting a tiger mosquito has a median of 0.0051, and a 90% credible interval of (0.0018, 0.0108). In other words, there is a 90% chance that for every 1000 cars on the road in this area in the summertime, between 2 and 11 are transporting tiger mosquitoes. A density plot of the posterior predictive distribution is shown in Fig. 2A.

### Sampling Efficiency Adjustment

These estimates are conservative in the sense that the vacuuming may not have detected all transported mosquitoes. In order to assess the probability of capturing tiger mosquitoes present in sampled cars, a preliminary test of sampling efficiency was performed under different waiting times and in-car environmental conditions (see Methods). We placed female tiger mosquitoes into a test car, leaving them for either 5 minutes or 10 minutes, with or without air-conditioning, and we used the same vacuuming technique as in the road and ITV sampling to try to recover each mosquito. Of 48 individuals placed in the car, 33 were recaptured (68.75%), 6 (12.5%) were observed escaping, and the remaining 9 (18.75%) were lost and are assumed to have escaped as well. Along with these recapture results, temperature and humidity inside the test car are indicated in Supplementary Table 3. If we adjust our main estimates based on this recapture rate by rescaling the posterior predictive distribution, we get a median of 0.0074 and a 90% credible interval of (0.0026, 0.0157).

In fact, the recapture rate may depend on specific conditions in the car. It was higher in the 10-minute test than in the 5-minute one, and highest when air conditioning was used. This could result from mosquitoes tending to rest on surfaces after longer waiting times and under harsher conditions, making them easier to collect. However, when we model these factors as independent variables in a logistic regression, we cannot confidently rule out that the observed differences result simply from random variation: modelled in a Bayesian framework using Hamiltonian Monte Carlo (HMC) sampling, large proportions of the posterior distributions of the coefficients on these factors are below zero (Supplementary Table 4); similarly, from a frequentist perspective, the standard errors of the maximum likelihood estimates for these coefficients indicate that none are statistically significant (p-values are all above 0.05, as can be seen in Supplementary Table 5).

### Evidence from Citizen Science

**Figure 3:**
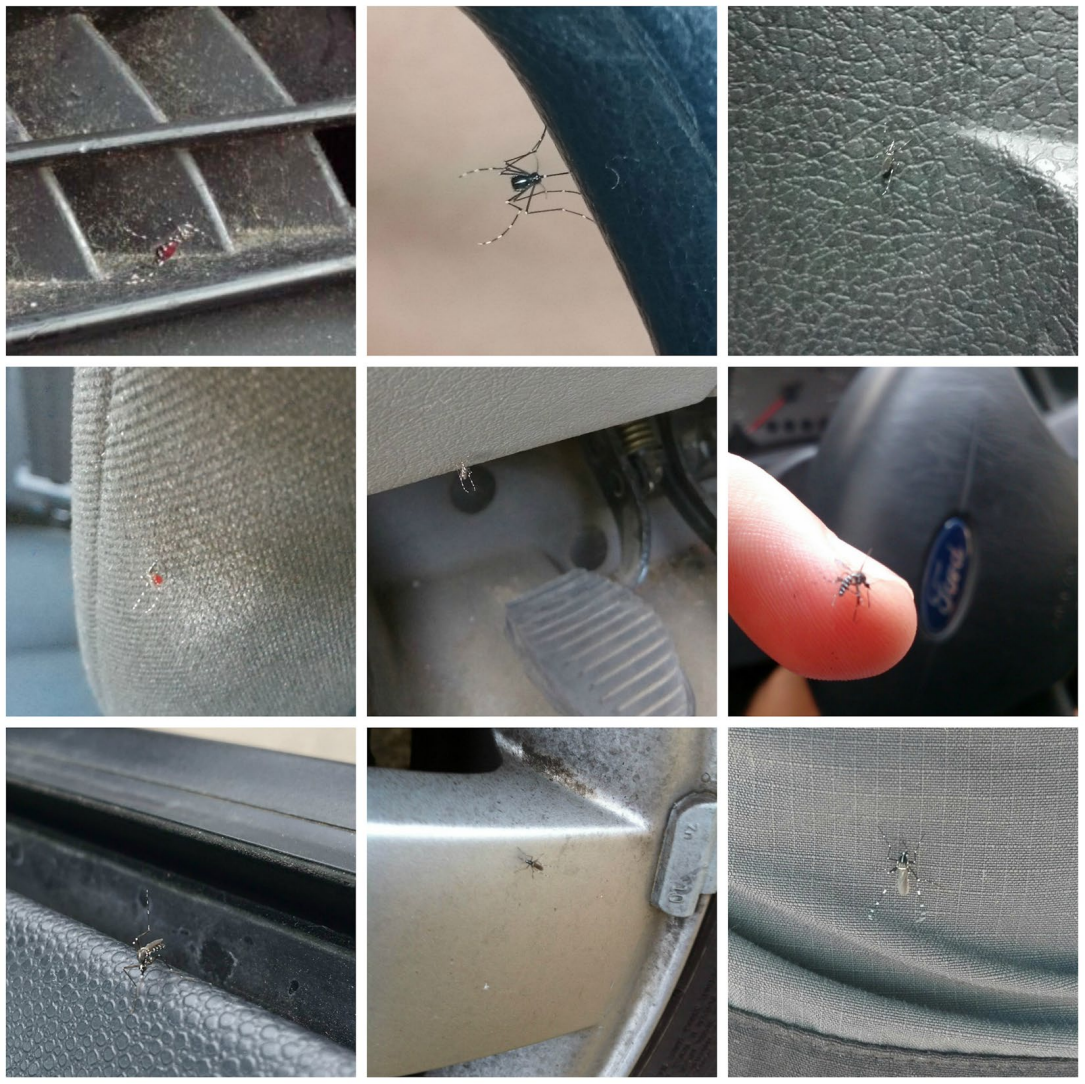
Photographs of tiger mosquitos in cars submitted by citizen scientists through *Mosquito Alert*.

Our results are corroborated by data gathered by citizen scientists and validated by entomologists during 2014–2016 using *Mosquito Alert*, a mobile phone application and web platform that helps the general public report tiger mosquito encounters^12,38^. We asked *Mosquito Alert* participants reporting adult tiger mosquitoes to indicate when their mosquito observations were inside vehicles. During 2014–16 we received a total of 98 reports in which the participant included a note suggesting that the reported mosquito had been discovered in a car or truck. Reports that included photographs were reviewed by a team of entomologists, which concluded that 11 possibly showed tiger mosquitoes, and 16 definitely showed tiger mosquitoes (examples are shown in Fig. 3). These 27 possible or definite tiger mosquito vehicle reports account for 1.3% of the total possible or definite tiger mosquito reports received during the 2014–16 period (2002 reports). We also administered a single-question survey to *Mosquito Alert* participants, asking them whether they had seen a tiger mosquito in their car during their last car trip. Of the 1673 people who responded, 386 (23%) answered “yes”, 1220 (73%) answered “no”, and 67 (4%) submitted a blank answer.

We do not take this *Mosquito Alert* data as a reliable indicator of the proportion of cars carrying mosquitoes. As described in the Methods section, we use the validated photographs as the basis for an informative prior in one version of the car transport model, but this is for purposes of robustness checking, and the results are identical to those without the citizen science information. We do, however, take these citizen science results as evidence, corroborating our car sampling results, that tiger mosquitoes are being transported in cars and that the numbers involved are not trivial. In addition, we use *Mosquito Alert* data on tiger mosquito prevalence in order to estimate inter-province mosquito transfers, as discussed below.

### Potential Inter-Province Mosquito Transfers

We take the car stop results as the best available estimate of the probability of a given car transporting a tiger mosquito, and we combine this with data on commuting patterns from Spain’s Economically Active Population Survey (APS) and data on tiger mosquito prevalence from *Mosquito Alert* to estimate potential inter-province flows of tiger mosquitoes in Spain. We find some evidence that car transport probability is positively associated with contemporaneous tiger mosquito prevalence in the municipality in which the car was parked overnight (see Fig. 2A,C, Supplementary Table 6): Modelled in a Bayesian framework as a logistic regression estimated with HMC sampling, with origin municipality *Mosquito Alert* ^12^ “alert probability” as an independent variable serving as a proxy for mosquito prevalence (see Methods), we find that 86% of the posterior distribution for the mosquito alert coefficient lies above zero (Fig. 2B). In other words, we estimate an 86% chance that mosquito transport is more likely for cars originating in municipalities with higher mosquito prevalence (as measured through alert probability). This leaves us with a relatively large amount of uncertainty: The posterior has a large variance and 14% lies below zero (Fig. 2B and Supplementary Table 6). Nonetheless, the result provides a reasonable basis to conclude that origin municipality prevalence matters, particularly given the inherent logic of this hypothesis. Figure 2C shows the posterior predictive interval across the full range of bi-weekly tiger mosquito alert probabilities used in the model.

**Figure 4:**
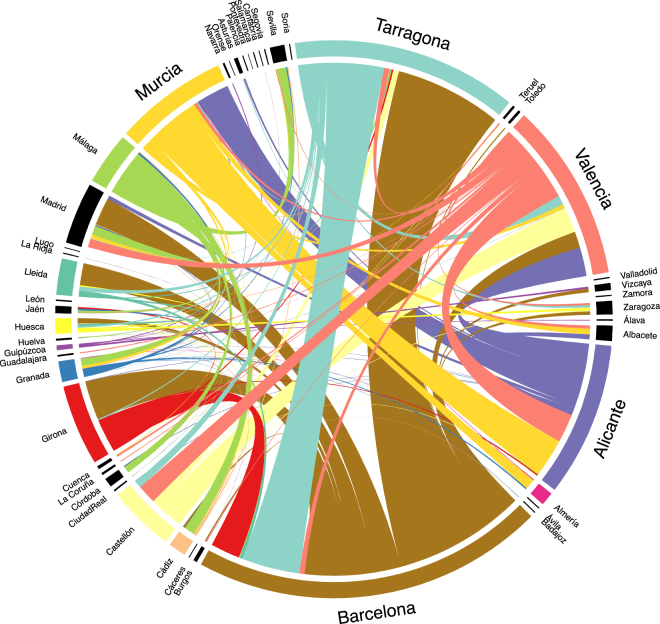
Relative densities of inter-province tiger mosquito transfers via commuter flows during September based on predicted probability of finding a mosquito in a car as a function of origin *Mosquito Alert* probability. Risk of transport out of municipalities in which tiger mosquito presence has not been confirmed is set to zero before aggregating to province level. Colours correspond to source province and link widths are proportional to transfer density. Provinces in which tiger mosquitoes have not been confirmed in any municipality have zero estimated outgoing transfers and are coloured black. Balearic Islands, Canary Islands, Ceuta, and Melilla are excluded. See also Fig. 6 in the Supplementary Information. Estimates based on commuter flow data and human-mosquito interaction data from 2014–2015.

Combining these model predictions with the APS commuting patterns as well as municipal population data (see Methods) allows us to estimate potential inter-province tiger mosquito flows for any given period of time. In Fig. 4 we report these estimates for September 2015, as this is the peak of the tiger mosquito season and a month in which the reported commuting patterns are more likely to be accurate (in contrast to August, when patterns may be more disrupted by vacations) while also close in time to the actual car-stop period.

**Figure 5:**
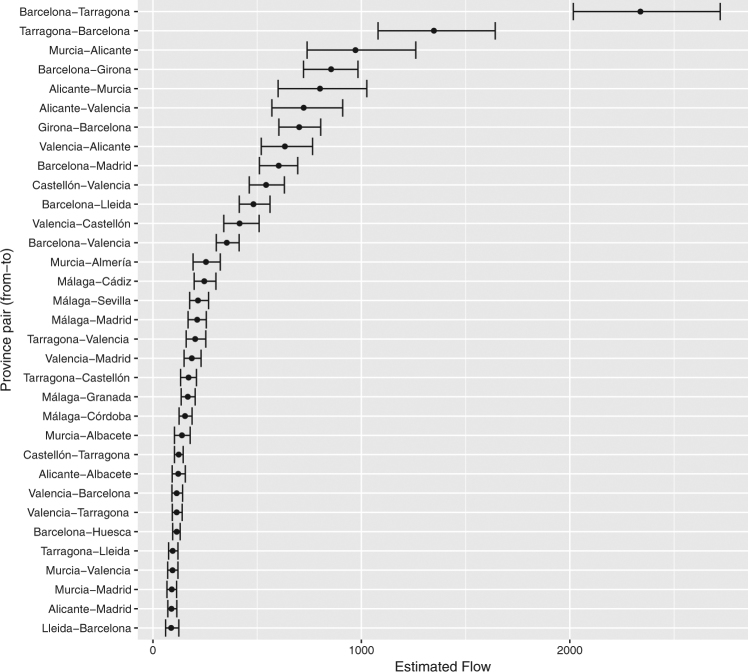
Estimated inter-province tiger mosquito transfers via commuter flows during September. Median estimates and 90% credible intervals based on predicted probability of finding a mosquito in a car as a function of origin *Mosquito Alert* probability. Risk of transport out of municipalities in which tiger mosquito presence has not been confirmed is set to zero before aggregating to province level. Province-pairs (y-axis) shown as origin-destination; plot shows only pairs with total September flows over 100 mosquitoes. Estimates based on commuter flow data and human-mosquito interaction data from 2014–2015.

Figure 5 shows the 90% credible intervals for these September predictions for all province pairs with predicted flows of over 100 mosquitoes. Although there is a relatively large amount of uncertainty as to the magnitude of the origin municipality mosquito alert coefficient in our model (Fig. 2B and Supplementary Table 6), this uncertainty becomes less important once it is combined with the commuter flow data and propagated up to the province-month level. Importantly, the patterns shown in Fig. 4, based on the medians of the posterior predictive distributions, are nearly identical to those calculated from the lower and upper values of the 90% credible intervals (Supplementary Figure 5).

**Figure 6:**
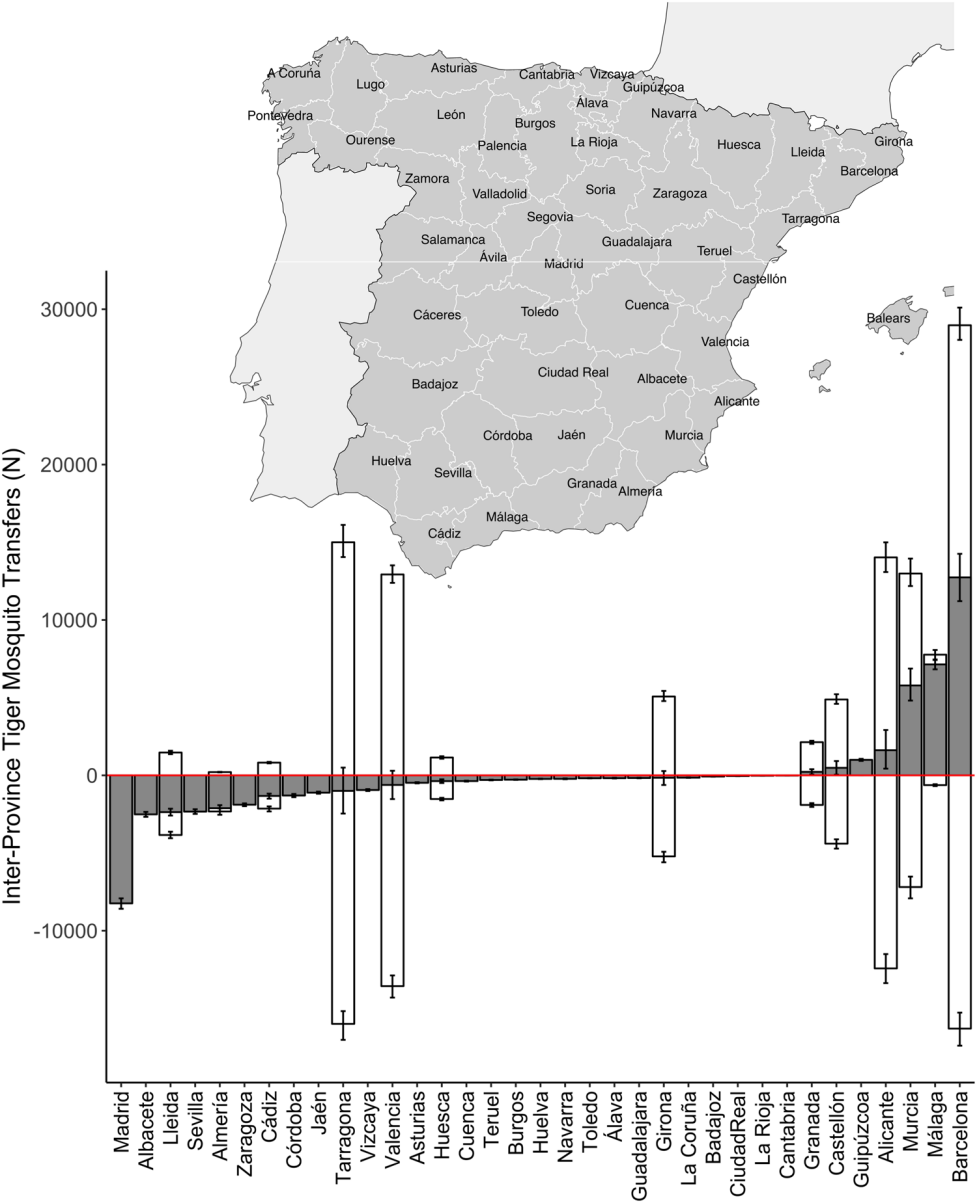
Potential sources and sinks of tiger mosquitoes in Spain by province. Grey bars show net annual potential tiger mosquito outflows (outflow – inflow); white bars show gross annual potential outflows as positive and gross annual potential inflows as negative. Error bars show 90% credible interval for each estimate. Provinces ordered by net outflow and map of Spanish provinces shown above bar plot for reference. Estimates based on commuter flow data and human-mosquito interaction data from 2014–2015. Map is own compilation, using Quantum GIS version 2.18 (http://www.qgis.org/), with Spain data (shapefiles) taken from the Spanish National Statistics Institute (INE) website: www.ine.es. Map also includes data from Eurostat, http://ec.europa.eu/eurostat. © EuroGeographics for the international administrative boundaries. Balearic Islands, Canary Islands, Ceuta, and Melilla are excluded from the analysis.

Figure 6 shows potential annual inflows, outflows, and net flows for all provinces that have some estimated inflow or outflow. We see that Madrid, Albacete, Lleida, Sevilla, Almería, and Zaragoza all have notable net inflows (negative grey bars), while Alicante, Murcia, Málaga, and Barcelona have notable net outflows (positive grey bars). On the other hand, focusing only on net flows masks the fact that some provinces, like Tarragona, Valencia, and Girona, have large gross inflows and outflows that simply offset each other in the net calculations. In this figure we also see from the small 90% credible intervals (vertical error bars) how the large predictive uncertainty in the transport risk model (Fig. 2B,C and Supplementary Table 6) becomes relatively small once combined with the commuter flows and aggregated – in this case to the level of province and year.

**Figure 7:**
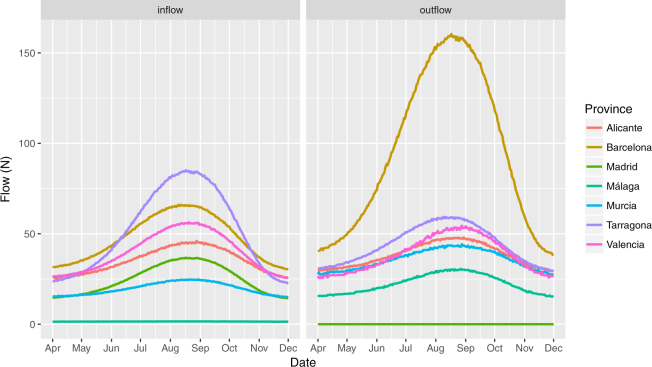
Median estimates of gross number of tiger mosquitoes transported per day into (left) and out of (right) selected provinces by commuters. Selected provinces are those with the highest annual tiger mosquito inflows and outflows. Estimates based on commuter flow data and human-mosquito interaction data from 2014–2015.

**Figure 8:**
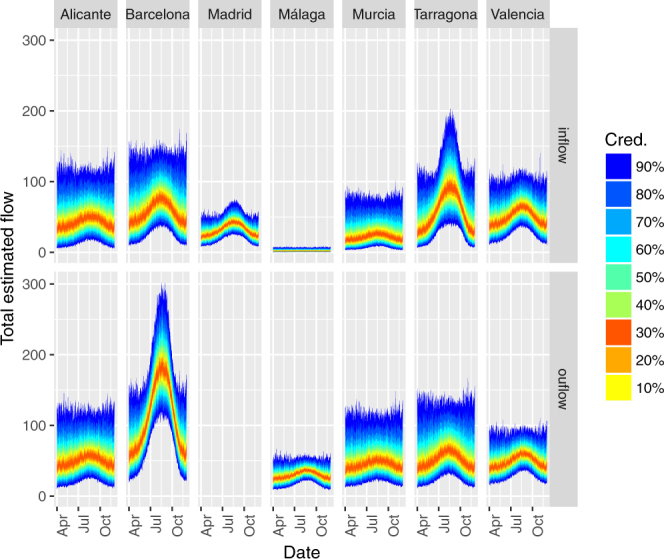
Credible intervals for gross number of tiger mosquitoes transported per day into (top row) and out of (bottom row) selected provinces by commuters. Selected provinces are those with the highest annual tiger mosquito inflows and outflows. Colours indicate credibility level. Estimates based on commuter flow data and human-mosquito interaction data from 2014–2015.

As the bi-weekly tiger mosquito alert probabilities show seasonal patterns, we also expect to see such patterns in the inter-province fluxes over time. Figures 7 and 8 show total estimated gross daily tiger mosquito inflow and outflow for the top source and sink provinces between March and December based on our model. Figure 7 relies on the medians of the posterior predictive distributions for each municipality-day, aggregated to the province level, while Fig. 8 captures uncertainty of daily flows by showing a range of credible intervals. (We show similar uncertainty plots for province pairs in Supplementary Figure 4). These curves show interesting patterns that are discussed below, but they should also be treated with caution because of the high amount of uncertainty in the estimates (Fig. 8) and because they assume constant commuting patterns and a constant relationship between tiger mosquito prevalence and transport probability–assumptions that are not likely to hold given summer holiday migration of humans and changes in diel activity patterns of mosquitoes.

## Discussion

The spread of the tiger mosquito worldwide and particularly in Europe has been notorious^39^, leading to widespread agreement that this mosquito is among the world’s most effective invasive species^40^. It has long been suspected that car transport plays a role in this process, but no direct testing had ever been performed on the road. Our work represents the first attempt to address this gap and quantify the impact of car-mediated transportation in the tiger mosquito’s invasive process.

Our main findings confirm that adult tiger mosquitoes are transported in cars, and suggest that for every 1000 cars on the road in the Barcelona area in the summertime, between 2 and 11 are carrying tiger mosquitoes. This implies that of the 6,528,979 car trips per workday in this area in the summer of 2015^41^, between 11,752 and 70,513 probably involved tiger mosquito displacements. These are conservative estimates considering that our preliminary sampling efficiency test suggests that only around 69% of the transported mosquitoes were likely to have been recovered. If we adjust our results based on sampling efficiency, we estimate that between 3 and 16 of every 1000 cars is carrying a tiger mosquito – or between 17,032 and 102,193 of the 6,528,979 daily car trips in the Barcelona area in the summer of 2015^41^.

Generalizing these results to the country level makes sense only if we take into account the mosquito prevalence in each car’s origin. Accounting for this based on citizen science data and combining this with province-level commuting data, we conclude that Barcelona is the province that exports the most tiger mosquitoes to other provinces. It is followed by Tarragona, Valencia, Alicante, and Murcia (Figs 4, 6–8). The daily flow curves in Figs 7, 8 suggest that Valencia starts the season with lower total exports than Murcia or Alicante, but then overtakes both of these provinces later on. This is consistent with the finding of Collantes *et al*.^42^ that tiger mosquitoes continue their outdoor reproductive activity during the winter in Murcia. We also see that of the provinces in which tiger mosquito presence has not yet been confirmed, Madrid has the largest potential inflows of car-transported tiger mosquitoes (Figs 6–8), based largely on flows from Barcelona, Valencia, and Málaga (Figs 4–6). Albacete, Sevilla, and Zaragoza are other non-confirmed provinces with notable inflows (Fig. 4). It is worth remarking as well that no comparison can be made from these data between the relative importance of the adult transport by cars and the immature transport within merchandise, as the latter has not been yet quantified.

Mosquito transport in cars is a rare event and modelling rare events is a challenge^43^. The potential for bias and large amounts of uncertainty hovers over any rare event prediction. Indeed, predicting the impact of cars on mosquito invasions is an even greater challenge than predicting long-distance dispersal by air or sea. This is so because car-mediated transport is an unpredictable dispersal mechanism with no specific directionality, presumably involving very few individuals at a time, through which the accumulation of rare events in large traffic flows can substantially redistribute mosquitoes at local and medium range scales, and even (though less frequently) over long-distances.

Car-mediated transportation is a combined process of a mosquito entering a car, surviving over time and distance, and then exiting the car. To better quantify mosquito fluxes, we need to better understand behavioural aspects of the interactions between mosquitoes, humans, and cars. We need to study temperature-related daily flight activity as well as the attraction distances and forces affecting mosquito presence in cars. For example, since storm drains that collect water on streets often harbour tiger mosquitoes^44,45^, cars parked on the street, rather than in a garage, should be more prone to transport tiger mosquitoes. Local landscape is highly heterogeneous in the metropolitan areas and may account for mosquito “hitchhike hotspots”, which should ideally be taken into account as a differential mosquito source to be managed^46^. We also need to assess survival probabilities in cars. During transport, mosquitoes may escape through open windows, be killed by occupants, or suffer desiccation and death due to air-conditioning^47^ or other conditions. As an example, 12.5% of the seeded mosquitoes in our preliminary tests were observed escaping, and 2 of the 4 mosquitoes captured in the field sampling were found dead.

Our results suggest that stratified dispersal involving adults hitchhiking in cars may be a key factor aiding tiger mosquito’s rapid expansion at medium scales, but conclusions about invasive capacity are not straightforward. It is clear that cars offer a great opportunity to colonize new areas but no estimates or modelling exist quantifying the minimum number of individuals that need to be successfully transported in order to establish the species in a new location. The establishment of a population in a new area depends on many other factors, such as habitat suitability at destination and the age and health status of the colonizers. Moreover, although our focus here is on the actual numbers of individuals transferred, another important consideration is gene flow and the creation of local subpopulations. Whether car transport patterns promote genetic mixing or whether first-established subpopulations act as buffers against newly invading genotypes is a question that deserves further attention^48^.

Another relevant knowledge gap is related to the high-resolution spatial and temporal variation in origin-destination human car mobility, and in particular, the frequency and timing of long-distance car trips (extreme statistics). In our mosquito flux modelling, we have assumed commuting patterns are constant across the season, which is clearly not the case. Of note, the seasonal trend shown in Figs 7, 8 is driven solely by the variation in *Mosquito Alert* probabilities at origin municipalities, not by variation in transport fluxes even though this latter variation must also exist. For example, commuting levels are most likely reduced and patterns changed by holidays during the summer period. In addition, tiger mosquito flight activity is temperature-related^49^, and hence the probability of a mosquito entering a car at rush hour, early morning (7–9 am) or late in the day (5–7 pm), could be biologically constrained in spring and autumn. In the latter months, mosquito hitchhiking behaviour may be restricted to midday hours, when traffic volume is lower.

We have provided data on an important phenomenon that, until now, has been taken for granted without any direct study. Nonetheless, more studies are needed to fully connect transportation with invasive success, accounting for factors related to life cycle completion within cars and at destination (e.g. habitat encounter and suitability). Our work illustrates both the complexity of measuring and accurately predicting mosquito transport by car, and its potential relevance for public health risk assessment. We hope that our findings will motivate further socio-entomological work on human mobility and car-related mosquito population dynamics and behaviour, and that this will contribute to control efforts to combat this disease-carrying species.

## Methods

### Car Sampling and Survey

To search for mosquitoes in passenger cars, sampling was performed in 7 locations in the Baix Llobregat region of Barcelona Province, Spain (Fig. 1): 3 road sites were located in roundabouts in the connection belts in motorways. The remaining 4 sites were the locations of Technical Vehicle Inspection Stations (ITVs). During 30 sessions of road sampling, from 16 July through 28 August 2015, a total of 358 cars were vacuumed for mosquitoes. A team formed by two entomologists accompanied two police officers who were carrying out routine car stops involving paperwork verification and security checks. The entomologists selected for vacuuming low occupancy 4-door sedan cars, with no colour preference (targeted cars). The process took less than 4 minutes per car and did not cause delay to the drivers because this was the time in which the police were anyway checking the car documents and driver’s license.

ITV sampling carried out in 29 sessions from 14 September through 23 October 2015 resulted in an additional 412 cars vacuumed. In total, 770 cars were examined during 106 hours of work (waiting times included). ITV sampling was performed in the line where cars were waiting for the technical checking. The general protocol was identical, except that at the time of vacuuming the driver had already opened the car door once to file paperwork as part of the inspection. The waiting time to enter the test booth was sometimes too short for complete processing, in which case the car had to be discarded from the sample.

In both the roadside and ITV sampling protocols, drivers were asked to volunteer to participate anonymously in the study. Refusals were recorded as such, with no other data collected. Drivers who volunteered to participate were asked about car features (brand, model, body and upholstery colour), use of air conditioning during the trip, location at which the car had spent the previous night (garage or street), municipality of origin and destination, estimated trip length at the time of the stop, and locations of intermediate stops. Volunteers were also asked about mosquito sightings and bites in the car during that day as well as on previous occasions. The ITV questionnaire was shorter and adapted to that specific situation. In accordance with Spanish law, no personal information of any kind was recorded.

#### Vacuuming protocol

All sampling operations were carried out by vacuuming with handheld entomological aspirators (Improved Prokopack Aspirator Model 1419, John W. Hock, Gainesville, Florida, US). These units are powerful (10.5 meter/sec airflow speed) yet lightweight thanks to a separate 12 V/12 A battery that is carried in a backpack and that allows ~4 hours of use before recharging is needed. Insects were collected in standard collection cups with a screw-mounted lid tightly fitted to the incoming vent.

We used vacuuming as the sampling protocol because it is the only methodology that works to sample mosquitoes in cars: other adult sampling tools (e.g. BG Sentinel traps or sticky traps) are passive, usually requiring 24 hours or more before collection, so they cannot be used in this context. The only other active sampling methods would be mouth aspiration, which would be much less efficient and more prone to heterogeneity in terms of handling than the vacuum aspirator. Indeed, active aspiration has long been used on a routine basis for checking planes and other vehicles for mosquitoes^50,51^.

The vacuuming protocol started by switching on the aspirator and then opening the left rear door of the car to check the backside and the underside of the front seats; the door pockets were then checked, followed by the floor carpet, the rear seats and the posterior windscreen. The protocol was repeated at the right rear side. Finally, the passenger front door was opened to check the front windscreen lower section, underparts of the front passenger seat, floor carpet as well as regions adjacent to the driver, such as the gear shift. For convenience, neither the driver’s seat nor any occupied seat were vacuumed. Only the door being used for vacuuming the respective section of the car was kept open, and it was immediately closed when finished. Operators were instructed not to check any personal items or make any remarks. Trunks were not checked due to privacy considerations and to avoid opening an additional door that might let in or out mosquitoes.

The sampling operator was well aware of the possibility of flying mosquitoes. The goal was to catch such mosquitoes in flight if possible, and to at least record their presence when observed. The grid of the collection cup was closely examined for mosquitoes without switching off the engine after every vacuuming operation; in case of presence of an individual, the cup was closed with its screw lid, labelled, and stored in a refrigerated container. The observed condition of the individual, location within the car, flight status and all relevant aspects of capture time were also recorded.

Captured mosquitoes were killed in the lab by deep-freezing at −22 °C for two hours, after which they were examined and identified to the species level. In the case of individuals found already inactive in the car, a basic analysis was carried out through visual examination to assess the condition of the individual. Several items were estimated on a 0–100% range as preservation indicators, such as the number of missing legs, integrity of the existing legs and antennae, preservation of both wings and of scutum scales, abdomen turgidity and overall flexibility.

### Sampling efficiency test

The purpose of the sampling efficiency test was to assess the probability of failing to detect tiger mosquitoes present in the sampled cars and the factors that might affect this. As the target type of car in the sampling protocol was a 4-door sedan, we used a 2008 Spanish version Ford Focus model for the sampling efficiency test. The test was carried out from 20–24 August 2015.

The test consisted of introducing, one at a time, insectary-reared female *Ae*. *albopictus* individuals through the left rear door of the car. Once the specific time for each test condition had elapsed, the car was vacuumed using the same standard protocol as used in the real sampling. The efficiency test ended when all areas had been vacuumed once, regardless of whether the introduced mosquito had been captured by then. As a result, each introduced mosquito could be recorded as either (i) captured, (ii) observed escaping through a door, or (iii) lost. The section of the car where each mosquito was captured was recorded, as well as the flying/resting status of the individual. Within each 5-minute sampling period, the probability of catching a mosquito in the car presumably changes with time but we did not consider the temporal dynamics of such a process. Instead, for each realization, we considered the overall efficiency of our sampling protocol just as we would assess the efficiency of a mosquito collector device. At the end of the test, a high intensity thorough vacuuming was performed to clean sweep every possible remaining mosquito. The car was located in a shadowed area of an urban environment; the tests were carried out between 10–14 h, which is the same time frame used in the real operations.

The factors studied were the time elapsed between introduction and vacuuming, and the air conditioning system inside the car. Three conditions were tested; (1) vacuuming after 5 minutes (N = 18), (2) after 10 minutes (N = 14) and (3) after 10 minutes with the air conditioning set at 25 °C on the car command panel (N = 16). Humidity and temperature were recorded inside the car using a Sato PC-5000 TRH digital termohygrometer (Sato Keiryoki Mfg. Co. KLtd, Tokyo, Japan) (see Supplementary Table 3). Treatment effects were modelled using logistic regressions fitted using HMC sampling in a Bayesian context with weakly informative prior distributions (Supplementary Table 4), as well as with maximum likelihood estimation (Supplementary Table 5). The Bayesian approach is reported for consistency with the other Bayesian models discussed here, but the results are straightforward from either a Bayesian or frequentist perspective. We implemented by the Bayesian approach with Stan^52,53^ using the rstanarm package for R^54,55^ with weakly informative Cauchy prior distributions with location 0 and scaling factor 2.5 (equivalent to a student-t distribution with 1 degree of freedom, mean of 0, and standard deviation of 2.5), as recommended by Gelman *et al*.^56^. We implemented the maximum likelihood estimates using the glm function in R^55^. Recapture success was regressed on a constant with no covariates, against the three treatment conditions, and against time and air-conditioning separately and jointly (Supplementary Tables 4–5).

### Citizen Science Data

The citizen science data discussed here comes from *Mosquito Alert* (previously called *Tigatrapp*), a scalable system that allows citizen scientists to report tiger mosquito encounters and provide other information through a mobile phone application, collecting data on a server where it is validated by experts and disseminated to public health agencies, mosquito control services, and the public^40^. The system helps participants correctly identify tiger mosquitoes, and observations include geolocation, key taxonomic information, optional photographs and optional notes. Citizen scientists’ photographs are validated in terms of species by a team of expert entomologists and double checked by a supervising senior entomologist. The *Mosquito Alert* mobile phone application also collects optional anonymous geolocation information from participants in order to estimate and adjust for sampling effort. The anonymization process assigns tracked locations to predefined sampling cells of 0.05 degrees latitude by 0.05 degrees longitude, and these are therefore the primary unit of analysis. Finally, *Mosquito Alert* facilitates surveys of participants and asks them to participate in “missions”, such as using a special hashtag in the notes section to mark reports of mosquitoes found in their cars.

The present analysis relies on both report and survey data from *Mosquito Alert*. Palmer *et al*.^12^ use the report data to estimate bi-weekly tiger mosquito alert probabilities across Spain. These are estimates of the probability of a reliable tiger mosquito report being sent by a citizen scientist from a given sampling cell during a two-week period, conditional on sampling effort, and we treat these as a proxy for relative tiger mosquito prevalence. We scale these estimates to the municipality level by resampling the posterior distributions for each sampling cell, with sampling weights given by the proportion of the municipality’s territory covered by each cell. In other words, for each municipality, we draw a sample of values from all of the posterior predictive distributions of the sampling cells that overlap that municipality. The probability of drawing each sample element from a given sampling cell is given by the proportion of the municipality’s territory covered by that cell. This is equivalent to drawing a spatially random sample of locations from within the municipality’s borders and evaluating the alert probability at each point.

The sampling cells in Spain, defined by latitude and longitude, range from 22–25 km^2^ when projected to a UTM grid. The Spanish municipalities are mostly larger, ranging from less than 1 to over 1,700 km^2^, with mean and median areas of 62 and 34 km^2^ (Supplementary Table 7, Supplementary Figure 3). The number of individual cells overlapping (in whole or part) each municipality ranges from 1 to 113, with a mean and median of 8 and 6. So most municipalities are overlapped by portions of at least 6 cells (see Supplementary Figure 1 for examples). Our municipality-level mosquito alert probability estimates are treated as proxies for mean tiger mosquito prevalence in each municipality, and they are used as covariates in the transport flux model discussed in the next section.

We also used the notes included with the *Mosquito Alert* reports to corroborate evidence that tiger mosquitoes travel in cars. In both 2014 and 2015 *Mosquito Alert* users were sent a mission asking them to add the text “#cotxe”, “#coche”, or “#car” (Catalan, Spanish, and English for “car”) to the notes of their reports when the reports were of adult tiger mosquitoes observed inside of cars. The first of these missions was released in late July 2014 and the second was released in September 2015. We report here the total number and proportion of reports received with a note containing at least one of these hashtags (case insensitive) or with at least one of the following words “cotxe”, “coche”, “car”, “camión”, “camió”, “camion”, “camio”, or “truck” (again, case insensitive).

Finally, we used the results of a *Mosquito Alert* survey in which participants were asked, “Did you notice any tiger mosquitoes in the car during your last car trip?” (“Has visto algun mosquito tigre dentro del vehículo en el último viaje que has realizado?” for users with devices set to Spanish; “Has vist algun mosquit tigre dins el vehicle en l'últim viatge que has fet?” for those with devices set to Catalan). Participants were able to answer by selecting either “yes” or “no” or selecting nothing.

### Car Transport Probability Model

We used the car stop data to fit a series of Bayesian models and make predictions about tiger mosquito flows across Spain. We treated the result of each stop as the realization of a random variable drawn from a Bernoulli distribution with probability $\theta .$ We estimated $\theta$ in two logistic regressions models, first a naive model with no covariates, and then a model with origin municipality bi-weekly tiger mosquito alert probability (discussed above). To keep the flux results conservative, we set the tiger mosquito alert probabilities to zero for all municipalities in which tiger mosquitoes have not yet been officially confirmed.

We fit the models using HMC sampling implemented by Stan^52,53^ using the rstanarm package for R^54,55^. For all estimated parameters we used weakly informative Cauchy prior distributions with location 0 and scaling factor 2.5 (equivalent to a student-t distribution with 1 degree of freedom, mean of 0, and standard deviation of 2.5), as recommended by Gelman *et al*.^56^. We also tried both models using informative priors, as recommended by Rainey^57^ as a way of improving and checking the robustness of the logistic regression estimates given the small number of positive observations: For the null model we gave the Cauchy distribution for the intercept a location of 0.013, based on the results from the citizen science hashtag mission; for the alert probability model, we gave the Cauchy distribution of the slope coefficient a location of 1. In both cases, the results with informative priors were nearly identical to those without, suggesting that the low number of positives does not bias the estimates here. We compared models using leave-one out cross validation (LOO) estimated with Pareto-smoothed importance sampling^58^. The primary comparison statistics were expected log pointwise predictive density (ELPD) and the LOO information criterion (LOOIC), which is analogous to the Akaike Information Criterion and is defined as −2*ELPD.

This approach is designed to deal with the small number of tiger mosquito occurrences in the data. Maximum likelihood estimation would yield biased results here^43^. Our Bayesian logistic regression models, fitted using Markov chain Monte Carlo sampling with weakly informative priors as well as informative priors, should be less susceptible to bias, and the fact that the two versions yield nearly identical results is encouraging^56,57^. Nonetheless, with only 4 captures, there is a high degree of uncertainty in the model estimates (Fig. 2B,C and Supplementary Table 6). We discuss this uncertainty further with respect to our flow estimates in the next section.

### Potential Inter-Province Flows

We predicted inter-province tiger mosquito flows by combining our estimates of car transport probability with commuter flow data drawn from the Spanish National Statistical Institute’s Economically Active Population Survey (APS)^59^ and its Continuous Municipal Register Statistics^60^. The APS includes a question on each respondent’s home province and work province, from which we built an origin-destination matrix of province-level commuting behaviour. We combined 8 iterations of this survey (2014–15) and estimated population-level counts using the sampling weights reported for each iteration. In total, we relied on 470,910 responses that include both home and work province. To combine this data with the municipality-level transportation risk estimates, we scaled the province-pair commuter flows down to the municipality level by calculating the outflows and inflows for each municipality as proportional to that municipality’s share of the total province population. The assumption is that inter-province commuters’ homes are distributed across municipalities in their home province similarly to the rest of the population’s homes in that province, and that inter-province commuters’ places of work are distributed across municipalities in their work province similarly to the rest of the population’s homes in that province. We estimated the municipal populations from the Continuous Municipal Register Statistics for January 2015^60^.

For purposes of mosquito transport estimation, we calculated total commuter flow from municipality X to municipality Y as the sum of the population living in municipality X and working in municipality Y (who could potentially transport mosquitoes from X to Y on their way to work) and the population living in municipality Y and working in municipality X (who could potentially transport mosquitoes from X to Y on their way home from work). Thus, we calculated the potential flow (M_ijk_) of tiger mosquitoes from municipality *i* to municipality *j* on day-of-year *k* as:

1 $${M}_{ijk}\,=({F}_{ij}+{F}_{ji})\,\ast \,{P}_{ik},\,\forall \,i\,\ne j$$

where *F*_*ij*_ is the commuter flow from province *i* to province *j*, *F*_*ji*_ is the commuter flow from province *j* to province *i*, and *P*_*ik*_ is the transport probability for province *i* on day-of-year *k*.

We are aware that commuting mobility is only a limited subset of the whole potential mosquito transport by car, and that we are missing vacation or leisure mobility, which mostly occurs during the high mosquito season. Thus, we treat our estimates as conservative and only as an initial step towards a more comprehensive understanding of the impact of passive mosquito mobility in the invasion process and on health risks.

In order to propagate the uncertainty of the transport model into the flow estimates, for each municipality-pair and each day of the year, we first estimated the posterior predictive distribution of the car transport probability for the origin municipality using the car transport probability model. We then drew a random sample of 400 values from this distribution and multiplied each by the inter-municipal commuter flow. We used this large vector of sample estimates as the basis for all aggregations by province, month, and year, maintaining the samples in each aggregation and then calculating relevant quantiles from them. Thus, we calculate the sample of daily flows for each province pair as:

2 $${P}_{abkl}=\sum _{i=1}^{I}\sum _{j=1}^{J}{M}_{ijkl},\,\forall \,i\,\ne j,\,a\,\ne b$$

Where ${P}_{{abkl}}$ is sample element *l* of the flow from province *a* to province *b* on day *k*, and ${M}_{{ijkl}}$ is sample *l* of the flow on day *k* from municipality *i* in province *a* to municipality *j* in province *b*. We calculated each element *l* in the sample of daily total outflows (*O*_*akl*_) for province *a* to the B other provinces in Spain to which *a* is connected by commuter outflows as the sum of ${P}_{{abkl}}$ over these *B* provinces:

3 $${O}_{akl}=\sum _{b=1}^{B}{P}_{abkl}$$

Similarly, we calculated each element *l* in the sample of total inflow (*I*_*bkl*_) on day *k* to province *b* from the *A* other provinces in Spain to which *b* is connected by commuter inflows as the sum of ${P}_{{abkl}}$ over these *A* provinces:

4 $${I}_{bkl}=\sum _{a=1}^{A}{P}_{abkl}$$

We calculated each element *l* in the sample of net outflow on day *k* from province *a* as:

5 $${N}_{akl}=\,{O}_{akl}-\,{I}_{akl}$$

Propagating the uncertainty into our flow estimates in this way shows that the relative importance of the uncertainty shrinks, relative to estimated flow size, when we combine the model estimates with the commuter flow information and aggregate our estimates to the level of provinces and months or years. Spain’s provinces range from 1,000 km^2^ to nearly 9,900 km^2^, with a bimodal distribution that has a mean and median area of 3,800 km^2^ and 2,000 km^2^ (Supplementary Table 7), much larger than the municipalities that serve as the basic unit of analysis in the car-transport probability model and the sampling cells for which mosquito alert probabilities are initially calculated (see Supplementary Table 7, and Supplementary Figure 2). Each province contains between 6 and 414 municipalities, with a mean and median 162 and 169.

The coefficient on the alert probability variable in the car transport probability model, fit at the level of municipalities and days, has a wide 90% credible interval of [−1.2, 6.9] (Fig. 2B and Supplementary Table 6). Once we combine this with the commuter flows and aggregate to the province level (Fig. 8, and Supplemental Fig. 4), we still see wide 90% credible intervals but seasonal patterns and differences between provinces and province-pairs are clear. Aggregating to the level of months and years further reduces the uncertainty, relative to the estimates (Figs 5, 6). This is because the commuter flow information is an important component of the estimate, and summing independent random variables (as we are doing in the aggregation) tends to increase the mean more than the standard deviation or inter-quantile ranges.

### Data and Code Availability

The data and code used in this analysis have been deposited with Zenodo and may be accessed at http://doi.org/10.5281/zenodo.838803 (data)^61^ and http://doi.org/10.5281/zenodo.838797 (code)^62^. In addition, the *Mosquito Alert* data and code, on which the car transport probability model draws, are available at https://doi.org/10.5281/zenodo.646531 (data)^63^ and https://doi.org/10.5281/zenodo.646576 (code)^64^. Daily snapshots of the *Mosquito Alert* data are available at http://doi.org/10.5281/zenodo.597466, and all of the code used for the *Mosquito Alert* mobile phone applications and server is available at https://github.com/MoveLab.

## Electronic supplementary material

Supplementary Information
