# Supplementary material for: Direct Evidence of Adult Aedes albopictus Dispersal by Car
Source: Sci Rep. 2017 Oct 24;7:14399. doi: 10.1038/s41598-017-12652-5 (PMC5656642; doi:10.1038/s41598-017-12652-5)
Supplement: Supplementary file 1 — Supplementary Information [file 41598_2017_12652_MOESM1_ESM.docx]

**Direct Evidence of Adult *Aedes albopictus* Dispersal by Car**

Roger Eritja^1,2,*,†^, John R.B. Palmer^1,3,4,†^, David Roiz^5^, Isis Sanpera-Calbet^2^, Frederic Bartumeus^1,3,6,*^

^1^CREAF, Cerdanyola del Vallès, Spain.

^2^Servei de Control de Mosquits del Consell Comarcal del Baix Llobregat, Sant Feliu de Llobregat, Spain.

^3^Centre d’Estudis Avançats de Blanes (CEAB-CSIC), Girona, Spain.

^4^Universitat Pompeu Fabra, Barcelona, Spain.

^5^MIVEGEC (Infectious Diseases and Vectors: Ecology, Genetics, Evolution and Control), Montpellier, France

^6^ICREA, Institut Català de Recerca i Estudis Avançats, Barcelona, Spain.

* Corresponding authors

^†^ These authors contributed equally to this work.

**SUPPLEMENTARY TABLES**

|  | **N sampled cars** | **% Declined** | **% AC** | **% Street night** | **Mean trip time / SD (min)** | **Captured females** | **Valid females** | **Live females** |
| --- | --- | --- | --- | --- | --- | --- | --- | --- |
| Road | 358 | 5.0 | 58.7 | 57.8 | 40 / 66.7 | 2 | 2 | 1 |
| ITV | 412 | 16.8 | 22.1 | 41.0 | 18 / 14.3 | 4 | 2 | 1 |
| Total | 770 | 13.2 | 39.1 | 48.8 | 27 / 47.9 | 6 | 4 | 2 |

**Supplementary Table 1**. Descriptive statistics of car sampling, including number (N) of sampled cars, percentage of drivers who declined survey and vacuuming, percentage of sampled cars that had been travelling with air-conditioning (AC), percentage of sampled cars that had been parked on the street, as opposed to in a garage, during the previous night, mean trip time (rounded to the nearest minute for clarity) plus standard deviation, and number and state of captured female tiger mosquitoes.

| **Location** | **Geo. ref. (Lon. Lat.)** | **Date** | **Species** | **Condition** | **Origin** | **Destination** | **Spent night** | **Trip time (min)** | **AC** |
| --- | --- | --- | --- | --- | --- | --- | --- | --- | --- |
| Road - Cornellà | 2.078322  41.35706 | July 22 | *Aedes albopictus* | Recently dead | Vallirana | Cornellà | Street | 20 | N |
| Road - Cornellà | 2.078322  41.35706 | July 27 | *Aedes albopictus* | Alive | St. Andreu de la Barca | Cornellà | Parking | 45 | N |
| ITV - Sant Just | 2.080425  41.39442 | Sept 17 | *Aedes albopictus* | Dead | Sant Feliu | NA | Parking | 3 | N |
| ITV- Cornellà | 2.078322  41.35706 | Sept 21 | *Aedes albopictus* | Alive | Hospitalet | NA | Parking | 10 | Y |
| ITV - Cornellà | 2.078322  41.35706 | Oct 1 | *Culex pipiens* | Alive | Viladecans | NA | Street | 15 | N |
| ITV - Sant Just | 2.080425  41.39442 | Oct 13 | *Aedes albopictus* | Alive | Molins de Rei | NA | Parking | 40 | Y |

**Supplementary Table 2:** Taxonomic conclusions about each of the 6 captured mosquitoes and survey responses from the drivers of the cars in which they were found. Note that the analysis relies only on the first 4 individuals in the table, as the 5^th^ was determined not to be a tiger mosquito and the 6^th^ was strongly suspected to have entered the car at sampling time.

| **Condition** | **N** | **Car Mean Temp (C)** | **Car Mean Hum (%)** | **% Captured** | **% Escaped** | **% Lost** | **% Flying** | **% Resting** |
| --- | --- | --- | --- | --- | --- | --- | --- | --- |
| 5 min | 18 | 32.0 | 47.7 | 61.11 | 22.22 | 16.65 | 90.9 | 9.1 |
| 10 min | 14 | 33.9 | 46.2 | 71.43 | 7.14 | 21.43 | 60.0 | 40.0 |
| 10 min + AC | 16 | 26.2 | 29.1 | 75.00 | 6.25 | 18.75 | 25.0 | 75.0 |

**Supplementary Table 3**. Conditions and results of preliminary test of sampling efficiency. For each experimental condition, N mosquitoes were introduced one-by-one into a car, which was then vacuumed. Mosquitoes not captured by the vacuuming were recorded as having escaped if they were observed exiting the car. All others were recorded as "lost". The percentages flying and resting refer to the observed activity of the captured mosquitoes at the moment when they were vacuumed.

|  | **Model** | | | | |
| --- | --- | --- | --- | --- | --- |
|  | **M0** | **M1** | **M2** | **M3** | **M4** |
| **Treatment B** |  | 0.4 |  |  |  |
|  |  | [-1.0, 1.8] |  |  |  |
| **Treatment C** |  | 0.6 |  |  |  |
|  |  | [-0.8, 2.0] |  |  |  |
| **Time (mins.)** |  |  | 0.1 |  | 0.1 |
|  |  |  | [-0.1, 0.3] |  | [-0.2, 0.4] |
| **AC** |  |  |  | 0.5 | 0.2 |
|  |  |  |  | [-0.8, 1.8) | [-1.3, 1.7] |
| **Constant** | 0. 8 | 0.5 | -0.0 | 0.7 | 0.0 |
|  | [0.2, 1.4] | [-0.4, 1.4] | [-1.9, 2.1] | [0.0, 1.4] | [-2.1, 2.1] |
| **N** | 48 | 48 | 48 | 48 | 48 |
| **ELPD** | -30.9 | -32.4 | -31.5 | -31.7 | -32.3 |
|  | (2.6) | (2.9) | (2.8) | (2.8) | (2.9) |
| **LOOIC** | 61.8 | 64.7 | 63.0 | 63.3 | 64.6 |
|  | (5.2) | (5.8) | (5.6) | (5.7) | (5.8) |
|  | | | | | |

**Supplementary Table 4:** Comparison of logistic regression models of preliminary test results in Bayesian framework. Recapture success is regressed on a constant with no covariates (M0), against treatment categories (A, 5 minutes with no air-conditioning is reference category; B is 10 minutes with no air-conditioning; C is 10 minutes with air-conditioning), and against time (M2) and air-conditioning (M3) separately and jointly (M4). All models fitted using Hamiltonian Monte Carlo (HMC) Markov chain sampling in a Bayesian context with weakly informative prior distributions. ELPD is expected log pointwise predictive density based on leave-one-out cross validation (LOO) estimated with Pareto-smoothed importance sampling. LOOIC is the LOO information criterion (analogous to AIC), defined as -2*ELPD. Estimated coefficients shown as posterior means with 90% credible intervals in brackets. Standard errors of ELPD and LOOIC estimates shown in parenthesis.

|  | **Model** | | | | |  |
| --- | --- | --- | --- | --- | --- | --- |
|  | **M0** | **M1** | **M2** | **M3** | **M4** |  |
| **Treatment B** |  | 0.464 |  |  |  |  |
|  |  | (0.764) |  |  |  |  |
| **Treatment C** |  | 0.647 |  |  |  |  |
|  |  | (0.753) |  |  |  |  |
| **Time (mins.)** |  |  | 0.112 |  | 0.093 |  |
|  |  |  | (0.127) |  | (0.153) |  |
| **AC** |  |  |  | 0.452 | 0.182 |  |
|  |  |  |  | (0.687) | (0.827) |  |
| **Constant** | 0.788^*^ | 0.452 | -0.108 | 0.647 | -0.012 |  |
|  | (0.311) | (0.483) | (1.051) | (0.372) | (1.134) |  |
| ***N*** | 48 | 48 | 48 | 48 | 48 |  |
| **Log Lik.** | -29.812 | -29.402 | -29.426 | -29.589 | -29.402 |  |
| **AIC** | 61.624 | 64.803 | 62.852 | 63.178 | 64.803 |  |
| ^*^p < .05; ^**^p < .01; ^***^p < .001  Standard errors in parentheses. | | | | | | |

**Supplementary Table 5:** Comparison of logistic regression models of preliminary test results in frequentist framework. Recapture success is regressed on a constant with no covariates (M0), against treatment categories (A, 5 minutes with no air-conditioning is reference category; B is 10 minutes with no air-conditioning; C is 10 minutes with air-conditioning), and against time (M2) and air-conditioning (M3) separately and jointly (M4). All models fitted using maximum likelihood estimation.

|  |  |  |
| --- | --- | --- |
|  | **M0** | **M1** |
| **Alert Prob.** |  | 2.6 |
|  |  | [-1.2, 6.9] |
| **Constant** | -5.3 | -6.7 |
|  | [-6.3, -4.5] | [-9.29, -4.67] |
| **N** | 770 | 770 |
| **ELPD** | -26.17 | -26.17 |
|  | (10.89) | (10.93) |
| **LOOIC** | 52.34 | 52.33 |
|  | (21.79) | (21.87) |

**Supplementary Table 6:** Comparison of logistic regression models of tiger mosquito car presence. Model M0 has no covariates while model M1 includes origin municipality tiger mosquito alert probability as an independent variable. Both models fitted using Hamiltonian Monte Carlo (HMC) Markov chain sampling in a Bayesian context with weakly informative prior distributions. ELPD is expected log pointwise predictive density based on leave-one-out cross validation (LOO) estimated with Pareto-smoothed importance sampling. LOOIC is the LOO information criterion (analogous to AIC), defined as -2*ELPD. Estimated coefficients shown as posterior means with 90% credible intervals in brackets. Standard errors of ELPD and LOOIC estimates shown in parenthesis.

|  | | | | | |
| --- | --- | --- | --- | --- | --- |
| Unit | Mean area | Median area | SD area | Min. area | Max. area |
| Province | 3,807 | 1,989 | 2,905 | 1,016 | 9,866 |
| Municipality | 61.56 | 34.49 | 92.26 | 0.03 | 1,754.83 |
| Sampling cell | 23.61 | 23.56 | 0.69 | 22.38 | 25.14 |

**Supplementary Table 7.** Summary statistics of province, municipality, and sampling cell areas in square kilometers.

**SUPPLEMENTARY FIGURES**

**
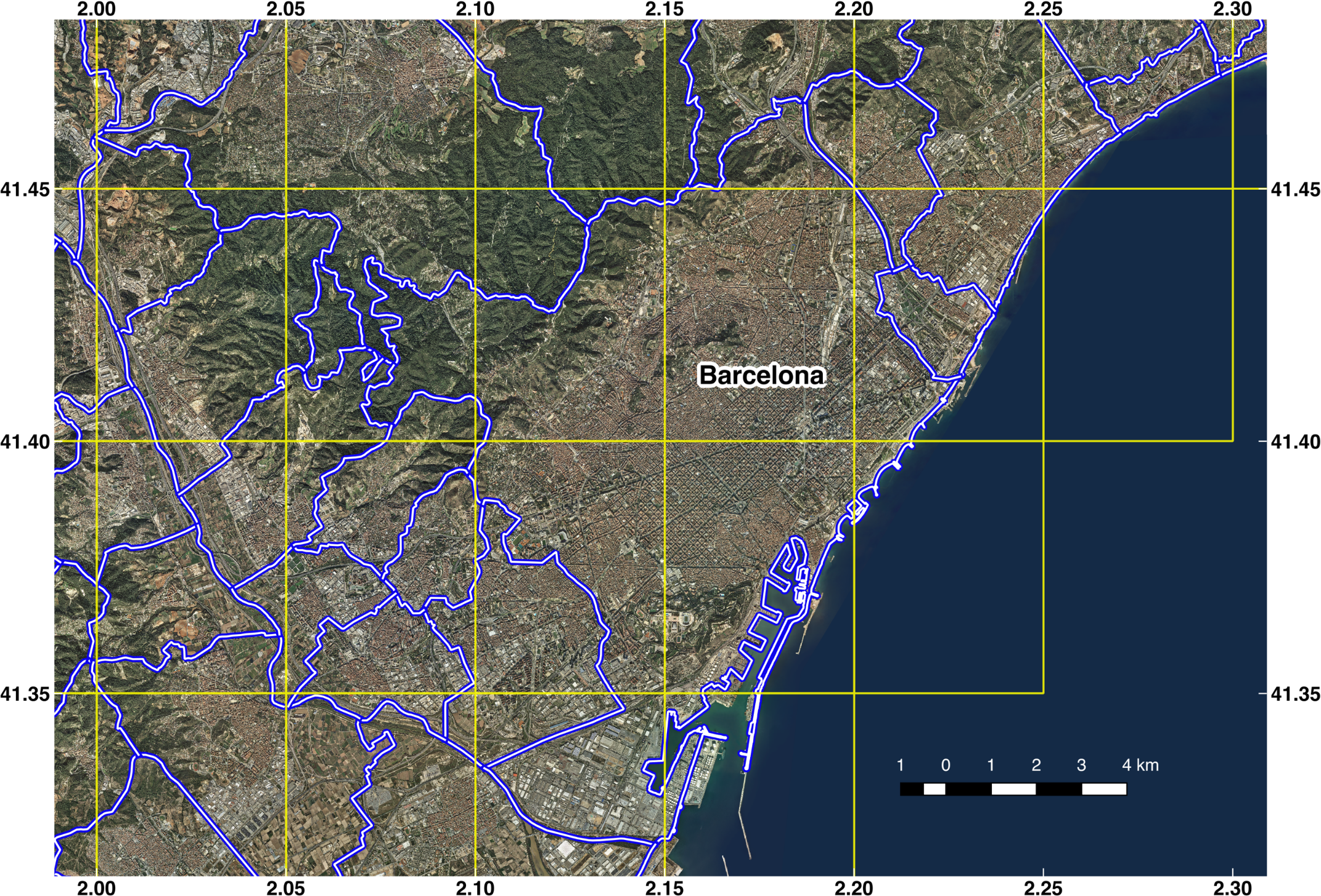
**

**Supplementary Figure 1**. Mosquito Alert sampling cells (yellow) and municipality boundaries (blue) in the Barcelona area, Spain. Barcelona municipality labelled; the other municipalities shown are all within Barcelona Province. Background image by the Cartographic and Geologic Institute of Catalonia, made available under CC-BY 4.0 International license at http://www.icgc.cat/Administracio-i-empresa/Descarregues/Imatges-aeries-i-de-satel-lit/Ortofoto-convencional. Map made using Quantum GIS version 2.18 (http://www.qgis.org/).


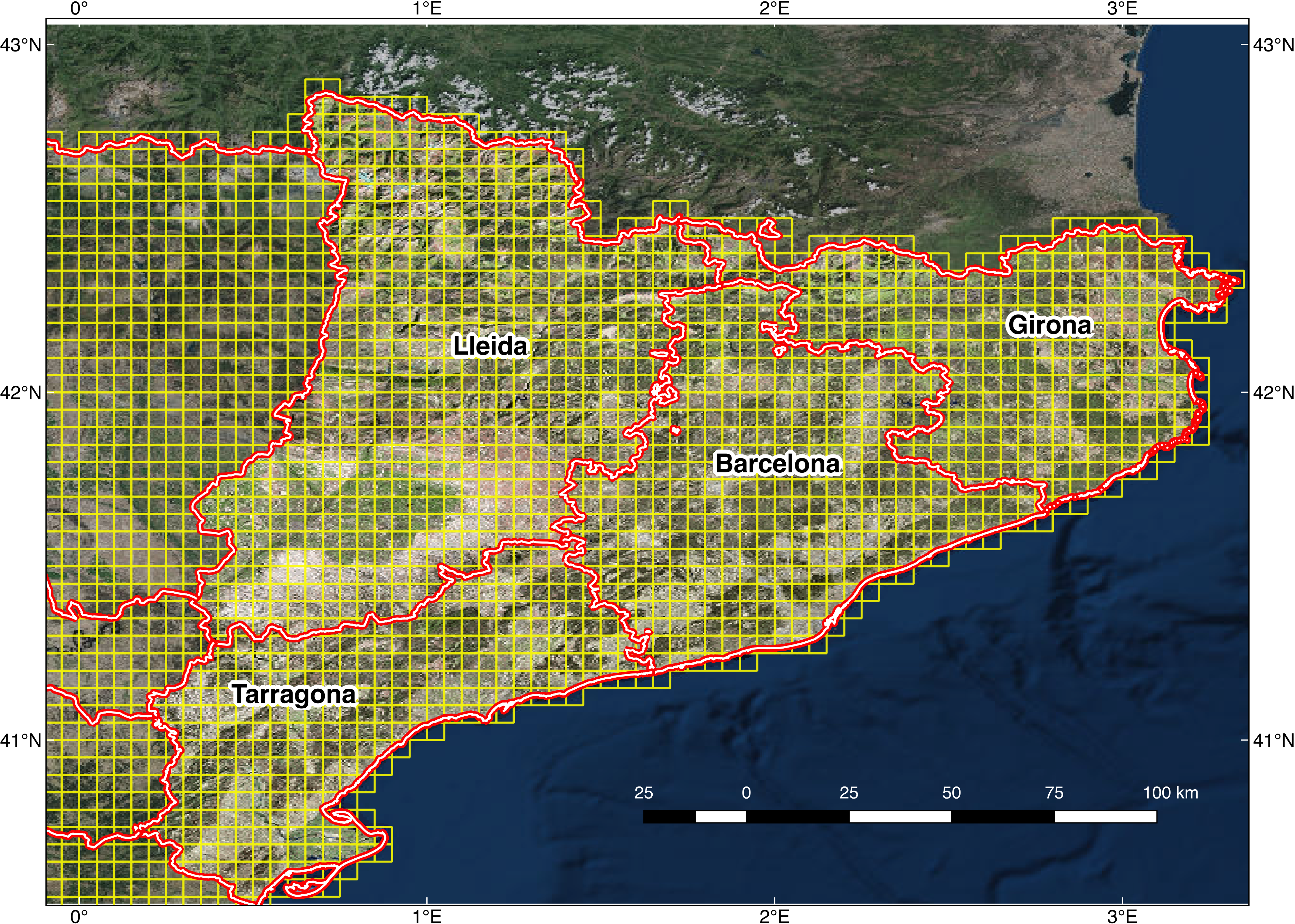


**Supplementary Figure 2.** Mosquito Alert sampling cells (yellow) and province boundaries (red) in Catalonia, Spain. Province names indicated. Background image by the Cartographic and Geologic Institute of Catalonia, made available under CC-BY 4.0 International license at http://www.instamaps.cat. Map made using Quantum GIS version 2.18 (http://www.qgis.org/).

**Supplementary Figure 3.** Density of Mosquito Alert sampling cell areas (yellow), province areas (red), and municipality areas (blue) in Spain. Densities scaled to have maximum of 1 for comparison. Log-scale used for x-axis to improve visualization.

**Supplementary Figure 4.** Credible intervals for number of tiger mosquitoes transported per day between selected province pairs by commuters. Selected province-pairs are those with the highest annual tiger mosquito inflows. Colours indicate credibility level and province-pair names shown at top as origin-destination. Estimates based on commuter flow data and human-mosquito interaction data from 2014-2015.

**Supplementary Figure 5.** Lower (**A**) and upper (**B**) limits of 90% credible intervals for relative densities of inter-province tiger mosquito transfers via commuter flows during September based on predicted probability of finding a mosquito in a car as a function of origin *Mosquito Alert* probability. Risk of transport out of municipalities in which tiger mosquito presence has not been confirmed is set to zero before aggregating to province level. Colours correspond to source province and link widths are proportional to transfer density. Provinces in which tiger mosquitoes have not been confirmed in any municipality have zero estimated outgoing transfers and are coloured black. Balearic Islands, Canary Islands, Ceuta, and Melilla are excluded. Estimates based on commuter flow data and human-mosquito interaction data from 2014-2015.
